# Importance of Using Sunscreen After Light or Laser Facial Treatment: A Literature Review

**DOI:** 10.3390/life15091484

**Published:** 2025-09-22

**Authors:** Kar Wai Alvin Lee, Lisa Kwin Wah Chan, Jong Keun Song, Cheuk Hung Lee, Jin-Hyun Kim, Kyu-Ho Yi

**Affiliations:** 1 EverKeen Medical Centre, Hong Kong, China; 2 Pixelab Plastic Surgery Clinic, Seoul, Republic of Korea; 3 You and I Clinic, Seoul 06001, Republic of Korea; 4 Division in Anatomy and Developmental Biology, Department of Oral Biology, Human Identification Research Institute, BK21 FOUR Project, Yonsei University College of Dentistry, Seoul 03722, Republic of Korea

**Keywords:** sunscreen, laser resurfacing, post-procedural care, aesthetic medicine, skin recovery, hyperpigmentation

## Abstract

Background: The application of sunscreen after light and laser facial treatments is vital for optimizing patient outcomes and minimizing complications. This review aims to highlight the importance of sunscreen use in post-procedural care within aesthetic medicine. Methods: A comprehensive literature search was conducted using keywords related to sunscreen application after light and laser treatments. Relevant studies involving human subjects and their effects on skin recovery were included. Results: The review found that early initiation of broad-spectrum sunscreen significantly enhances skin recovery and reduces inflammation following laser procedures. Sunscreens containing physical blockers, such as zinc oxide and titanium dioxide, were identified as the most effective in preventing sensitization and irritation. Patient education regarding sun protection practices was emphasized, revealing a gap between knowledge and adherence among patients. The inclusion of specialized formulations with anti-inflammatory properties showed promise in mitigating post-inflammatory hyperpigmentation. Conclusions: While the current literature underscores the critical role of sunscreen in post-laser care, further research is necessary to explore long-term effects and develop innovative formulations. Clinicians should prioritize patient education and adherence to sunscreen protocols to maximize the benefits of light and laser treatments and enhance overall patient satisfaction.

## 1. Introduction

Light and laser facial treatments have gained immense popularity in dermatology and aesthetic medicine due to their effectiveness in addressing various skin concerns such as wrinkles, scars, pigmentation, and overall skin rejuvenation [1,2,3,4,5,6,7,8]. These non-invasive procedures use focused light energy to target specific skin layers, promoting collagen remodeling and enhancing skin appearance [9,10]. While laser therapies can yield significant cosmetic improvements, they also alter the skin’s integrity, making it more vulnerable to external stressors, particularly ultraviolet (UV) radiation [1,11].

Following laser treatments, the skin undergoes a healing process characterized by increased sensitivity and a temporary impairment of its natural barrier function [12,13]. This compromised state heightens the risk of photodamage, which can lead to adverse effects including erythema, hyperpigmentation, and prolonged recovery times [14,15]. Consequently, the importance of adhering to a comprehensive post-procedure care regimen cannot be overstated. Among the most critical elements of this regimen is the application of sunscreen [16,17].

Sunscreens serve as a protective barrier against harmful UV rays, which can exacerbate the skin’s sensitivity post-treatment and negate the benefits achieved through laser therapy [14,18]. Research highlights that patients who diligently use broad-spectrum sunscreens following their procedures experience better healing outcomes, reduced incidence of complications, and longer-lasting results [19,20]. Furthermore, educating patients about the significance of sun protection not only fosters compliance but also empowers them to take an active role in their skin care [21,22].

This introduction aims to outline the pivotal role of sunscreen in the post-laser treatment context. By examining current literature and evidence-based practices, this review will emphasize the need for standardized patient education on sun safety. Ultimately, understanding and implementing effective sunscreen protocols can enhance treatment effectiveness, safeguard skin health, and improve overall patient satisfaction in aesthetic dermatology.

## 2. Materials and Methods

This study is a narrative review aimed at synthesizing evidence from various clinical studies and expert opinions without applying the rigid inclusion and exclusion criteria of a systematic review. A comprehensive literature search was conducted in MEDLINE, PubMed, and Ovid databases, utilizing predefined keywords (“Sunscreen,” “Sunblock,” “Skin Protection,” “Light Therapy,” “Laser Treatment,” and “Laser Therapy”) combined with Boolean operators (AND, OR) to refine the search. The search was limited to studies published in English between January 2000 and January 2024, focusing on clinical trials, case studies, original research articles, and expert consensus papers related to laser therapy and sunscreen use. The studies selected were categorized according to their level of evidence using the Oxford Centre for Evidence-Based Medicine hierarchy, ranging from high-level systematic reviews to expert opinions [23]. Studies were included if they addressed sunscreen use following ablative or non-ablative laser treatments, Intense Pulsed Light (IPL), or other light-based therapies. Studies were excluded if they did not focus on post-laser sunscreen use, were not in English, or lacked sufficient methodological detail. Reference lists of relevant articles were also screened to identify additional eligible studies.

## 3. Results

### 3.1. Ablative Laser Treatment and Sunscreen Outcomes

#### 3.1.1. Sunscreen After Ablative Laser Treatment

The incidence of post-inflammatory hyperpigmentation (PIH) and prolonged erythema following ablative laser procedures has been highlighted in multiple studies. Fulton et al. [24] demonstrated that immediate broad-spectrum sunscreen after CO_2_ resurfacing reduced PIH rates from 38% in untreated controls to 22% in the sunscreen group at eight weeks (Level III). Weinstein [25] similarly reported that patients undergoing Er:YAG resurfacing who used sunscreen twice daily experienced shorter erythema duration, with a mean reduction of 5 vs. 9 days, and fewer pigmentary changes compared to those without photoprotection (Level IV). These early observations established the principle that photoprotection directly reduces common complications after ablative procedures.

Additional reports reinforced this protective effect. Levy et al. [26] and Alster et al. [27] described decreased dyschromia and faster epithelial recovery among patients instructed to use sunscreen regularly following resurfacing (Level IV). Chen et al. [28] and Lee et al. [29] provided further evidence in Asian cohorts, noting reductions in PIH between 25–40% at 12 weeks, particularly in Fitzpatrick skin phototypes IV–VI (Level IIb). More recently, Wanitphakdeedecha et al. [30] confirmed these findings in a prospective setting, demonstrating consistent reductions in pigmentary relapse among patients using photoprotection after fractional CO_2_ laser (Level IIb). Collectively, evidence ranging from case series to prospective cohorts demonstrates that sunscreen is a low-cost, effective intervention for reducing pigmentary complications and improving overall recovery after ablative resurfacing.

Beyond individual trials, expert consensus and guideline statements emphasize that photoprotection should be initiated as soon as the epidermal barrier is adequately restored [29,31]. Regular reapplication throughout the day is recommended to maintain continuous coverage, and tinted or iron oxide-containing formulations may offer additional benefit by protecting against visible light, which has been implicated in pigmentary relapse, particularly in darker skin types [32,33]. Although much of this guidance is derived from lower-level evidence and expert opinion (Level III–IV), it reflects broad clinical agreement that sunscreen is not merely an adjunct but a core component of standard post-ablative management.

#### 3.1.2. Hydroxyapatite-Based Sunscreen After Laser Ablation

Experimental evaluation of hydroxyapatite-containing sunscreen has suggested enhanced ultraviolet attenuation and favorable tolerability in post-laser skin. Clinical testing demonstrated acceptable safety profiles without delaying wound healing, while in vitro findings indicated superior UV-scattering compared with conventional formulations [32,33]. Although evidence remains limited and largely preclinical, these results highlight the potential utility of hydroxyapatite-based sunscreens as adjunctive photoprotection after ablative procedures (Level Ib).

### 3.2. Non-Ablative Laser Treatment and Sunscreen Outcomes

Non-ablative procedures, including fractional resurfacing and superficial chemical peels, have also been shown to benefit significantly from consistent sunscreen use. Madnani et al. [17] documented that patients who initiated sunscreen early and reapplied as instructed experienced reduced post-procedure pigmentation and faster recovery. Puaratanaarunkon and coworkers [31] observed that regimented sunscreen use improved short-term recovery and lowered the incidence of pigmentary changes in non-ablative laser patients (Level IIII). Similarly, Zelickson and coworkers [33] and Alster and colleague [14] reported better healing trajectories and reduced dyschromia in patients adhering to structured photoprotection (Level IV). These consistent findings underscore the importance of broad-spectrum sunscreen use in minimizing adverse effects after non-ablative interventions.

Additional reports have emphasized practical considerations in product choice. Callender et al. [32] highlighted the importance of recommending broad-spectrum formulations that are well tolerated in skin of color, with tinted or iron oxide-containing sunscreens providing added visible-light protection (Level IV). Such formulations are particularly useful for patients at risk of melasma or PIH relapse. In line with these findings, recent guidelines advocate for barrier-friendly products and scheduled re-application to optimize adherence and clinical outcomes. Table 1 summarizes sunscreen ingredients most often reported to have anti-inflammatory or barrier-stabilizing properties, together with their clinical implications, and serves as a practical reference for clinicians when advising patients on sunscreen selection [34,35,36,37,38].

### 3.3. Intense Pulsed Light (IPL) Treatment and Sunscreen Outcomes

Several studies have examined the role of sunscreen in optimizing outcomes after IPL treatment. Ruvolo et al. [44] reported that consistent sunscreen use following IPL was associated with significantly lower recurrence of melasma at six months (28% vs. 47% in controls) (Level IIb). Amici et al. [16] observed that patients treated with IPL who adhered to structured photoprotection protocols experienced better erythema control and greater satisfaction than those who were less compliant (Level III). Additional observational data further support that regular sunscreen application enhances treatment durability and minimizes post-inflammatory pigmentary changes in patients undergoing IPL (Level IV).

Collectively, these findings indicate that adherence to broad-spectrum sunscreen after IPL procedures reduces relapse of pigmentary disorders and improves overall cosmetic outcomes. While much of the evidence remains observational, the consistency of results across different cohorts underscores the importance of integrating structured photoprotection into post-IPL aftercare.

### 3.4. Light-Based Treatments for Specific Purposes

#### 3.4.1. Sunscreen to Prevent Adverse Effect of Light Treatment

Observational studies and clinical surveys have shown that inadequate photoprotection after light-based treatments increases the risk of adverse outcomes, particularly in patients with skin of color. Callender et al. [32] emphasized the importance of broad-spectrum formulations that are cosmetically acceptable and well tolerated in darker phototypes, highlighting the role of tinted or iron oxide-containing sunscreens in blocking visible light (Level IV). These recommendations are echoed by other expert groups, who noted that simplified instructions and structured patient counseling improved adherence and reduced pigmentary complications after treatment (Level IV).

#### 3.4.2. Sunscreen After Using Light Treatment to Treat Melasma

Photoprotection is a critical component of maintaining the benefits of light-based interventions for melasma. Passeron et al. [45] demonstrated that strict sunscreen use, especially with products providing visible-light protection, significantly reduced relapse rates in patients at high risk of recurrence (Level IIb). These findings are consistent with broader evidence supporting sunscreen as a cornerstone of melasma management, not only for preventing exacerbation but also for sustaining therapeutic gains after procedures (Level IIb–III).

### 3.5. Sunscreen Formulation

Studies included in this review described a range of sunscreen formulations used after dermatologic procedures. Most commonly, broad-spectrum mineral filters such as zinc oxide and titanium dioxide were employed, either alone or in combination with organic filters to improve cosmetic acceptability and photostability [46,47]. Several products incorporated additional antioxidants or anti-inflammatory agents, which were reported to mitigate erythema and oxidative stress during recovery (Level IV). Tinted formulations containing iron oxide were highlighted by Callender et al. [32] and Passeron et al. [45] as particularly valuable for patients with skin of color or those at risk of melasma relapse, due to their added protection against visible light (Level IV–IIb). Collectively, these findings suggest that the choice of sunscreen after laser and light-based procedures should balance broad spectral coverage, tolerability on healing skin, and ease of reapplication to maximize adherence and outcomes.

## 4. Discussion

In this narrative review, we summarized current evidence on the role of sunscreen in post-procedural care following laser treatments. Earlier studies consistently showed that sunscreen reduces post-inflammatory hyperpigmentation (PIH), erythema, and delayed healing. Fulton Jr. et al. [24] and Weinstein et al. [48] demonstrated that photoprotection after ablative procedures minimized these complications, while Puaratanaarunkon et al. [31] reported that broad-spectrum sunscreens with anti-inflammatory components provided additional benefit in reducing PIH. These findings established sunscreen as a core element of post-laser management, even though much of the evidence came from case series and observational data (Level IV).

More recently, higher-level studies have reinforced and expanded these observations. Tran et al. [49] conducted a randomized controlled trial showing that immediate sunscreen use after ablative laser significantly reduced PIH, while Xu et al. [50] confirmed in a systematic review and meta-analysis that structured photoprotection improves recovery outcomes and patient satisfaction. In populations with skin of color, López et al. [51] and Tuchinda et al. [52] highlighted the added role of tinted or iron oxide-containing formulations in preventing visible-light-induced pigmentary relapse. Together, these findings indicate that sunscreen after dermatologic procedures is now supported not only by expert consensus and case reports, but also by high-quality randomized and pooled evidence.

### 4.1. Importance of Sunscreen Use Post-Laser Treatment

The risk of adverse effects such as post-inflammatory hyperpigmentation (PIH), erythema, and delayed healing is markedly increased after laser treatments. Fulton Jr. et al. [24] emphasized that adherence to sun protection protocols is essential for minimizing complications, and Weinstein et al. [48] also reported better outcomes in patients who consistently applied sunscreen after ablative procedures. Puaratanaarunkon et al. [31] further suggested that broad-spectrum sunscreens with anti-inflammatory agents provide additional benefit in reducing PIH compared with standard formulations. Although these studies largely represent Level IV evidence, they collectively highlight the central role of sunscreen in post-laser care. More recently, higher-level evidence has strengthened this conclusion: Tran et al. [49] conducted a randomized controlled trial showing that immediate initiation of sunscreen after ablative resurfacing significantly reduced PIH (Level Ib), while Xu et al. [50] confirmed through a systematic review and meta-analysis that structured photoprotection consistently improves recovery, reduces dyschromia, and enhances patient satisfaction (Level Ia).

Early initiation of sunscreen and careful formulation are critical in optimizing recovery. Wanitphakdeedecha et al. [30] reported that patients who began sunscreen use on the first day after fractional ablative resurfacing experienced faster re-epithelialization and reduced inflammation. Chen et al. [28] and Lee et al. [29] demonstrated that ablative and non-ablative procedures increase skin permeability, raising concerns of systemic absorption of topical products. This finding necessitates careful product selection, with physical blockers such as zinc oxide and titanium dioxide generally preferred in the immediate post-treatment phase because of their safety and tolerability on compromised skin [14,53]. Balancing efficacy, safety, and cosmetic acceptability remains a practical challenge in clinical care.

Patients with darker phototypes (Fitzpatrick III–VI) are at particularly high risk of PIH, which tends to be more persistent and cosmetically concerning due to heightened melanogenic activity and stronger inflammatory responses [24,30,53]. Tinted sunscreens, especially those formulated with iron oxides, provide substantial protection against visible light, including high-energy blue light, which standard untinted sunscreens often fail to block [45]. By attenuating visible light penetration, these formulations can significantly reduce the risk of PIH in skin of color while also improving cosmetic acceptability by covering erythema and dyschromia, thereby encouraging adherence. López et al. [51] emphasized their role in reducing pigmentary relapse in patients with skin of color, highlighting that tailored product choice is key to effective photoprotection.

Visible light, especially high-energy blue light (400–500 nm), has emerged as a critical factor in pigmentary relapse. It penetrates deeper into the dermis than UVB and induces melanogenesis through oxidative stress pathways. Mechanistically, visible light—particularly high-energy blue light—can activate the photoreceptor opsin-3 in human melanocytes. This activation triggers a signaling cascade involving increased intracellular calcium and cyclic AMP levels, leading to tyrosinase upregulation and enhanced melanin synthesis [51,52]. Such opsin-3-mediated pathways contribute to prolonged pigmentation in melanin-rich skin even after the initial inflammatory phase has resolved. Tuchinda et al. [52] highlighted that these mechanisms explain the persistent pigmentation seen in skin of color following procedures, reinforcing the importance of visible-light protection.

Consensus guidelines and expert recommendations now identify tinted sunscreens containing iron oxides, often combined with topical antioxidants such as vitamins C and E or ferulic acid, as best practice for post-procedure photoprotection, particularly in patients with skin of color [30,44]. Iron oxides effectively block visible light, while antioxidants neutralize reactive oxygen species generated by both UV and visible light exposure. Together, these strategies provide synergistic protection against pigmentary relapse, support faster recovery, and enhance long-term aesthetic outcomes. Incorporating these approaches into routine practice ensures that sunscreen use after laser treatments is not only evidence-based but also tailored to diverse patient populations.

### 4.2. Types of Sunscreens and Their Efficacy

The selection of appropriate sunscreen formulations is paramount to optimizing post-procedural outcomes. Puaratanaarunkon and coworkers [31] reported that broad-spectrum sunscreens containing anti-inflammatory agents were more effective in reducing post-inflammatory hyperpigmentation (PIH) following picosecond laser treatments compared with standard formulations (Level IV). Their findings suggest that incorporating such advanced formulations into post-procedure care can enhance both efficacy and patient satisfaction. More robust evidence has since reinforced these observations. Xu et al. [50], in a systematic review and meta-analysis (Level Ia), confirmed that consistent use of broad-spectrum photoprotection reduces adverse pigmentary outcomes and accelerates recovery across a variety of dermatologic procedures.

Formulation choice is also critical for patient safety. Boonchai and colleagues [53] reported that certain chemical sunscreen agents may provoke allergic or irritant reactions in post-resurfacing skin, highlighting the importance of physical filters such as zinc oxide and titanium dioxide, which are generally well tolerated in compromised skin. López et al. [51] further emphasized that tinted sunscreens containing iron oxides provide not only improved visible-light protection but also better cosmetic acceptability, which is especially relevant in patients with skin of color at high risk of PIH. This is supported by Tuchinda et al. [52], who demonstrated the role of visible-light-induced melanogenesis and the efficacy of iron oxide formulations in mitigating this pathway.

Together, these findings indicate that sunscreen recommendations after laser and light-based procedures should be individualized, balancing efficacy, safety, and cosmetic acceptability. Current evidence supports the preferential use of physical or tinted formulations, ideally supplemented with antioxidant protection, to minimize complications and improve long-term outcomes.

### 4.3. Patient Education and Compliance

Patient education is a critical determinant of successful photoprotection after dermatologic procedures. Earlier studies reported that even when sunscreen was prescribed, many patients applied it inconsistently or inadequately, limiting its protective benefit [24,30,53]. Barriers included cosmetic dissatisfaction, misconceptions about necessity, and the perceived complexity of application regimens. These findings underscore that structured education and simplified instructions are essential to improve adherence.

More recent evidence confirms that compliance is a key factor influencing outcomes. Xu et al. [50], in a systematic review and meta-analysis, identified poor adherence as a major contributor to heterogeneous results across clinical trials. López et al. [51] further emphasized that tinted sunscreens enhance both visible-light protection and cosmetic acceptability, thereby improving adherence in patients with skin of color. Collectively, these data indicate that sunscreen efficacy depends not only on product selection but also on patient behavior, and clinicians should prioritize clear counseling, individualized product recommendations, and follow-up to ensure consistent use.

### 4.4. Practical Clinical Recommendations for Post-Laser Photoprotection

Practical recommendations for sunscreen use after laser and light-based procedures must balance efficacy, safety, and adherence. Earlier studies emphasized that initiating sunscreen promptly after re-epithelialization reduces the risk of post-inflammatory hyperpigmentation (PIH), erythema, and delayed healing [24,30,31]. In the immediate post-procedure phase, when the barrier is still compromised, physical sunscreens containing zinc oxide or titanium dioxide are generally favored for their safety profile [14,53]. These formulations minimize irritation and are less likely to provoke allergic reactions compared with certain chemical agents. More recently, higher-level evidence has confirmed the importance of early initiation. Tran et al. [49], in a randomized controlled trial, demonstrated that immediate sunscreen application after ablative resurfacing significantly reduced PIH compared with delayed use (Level Ib). Xu et al. [50], in a systematic review and meta-analysis, similarly concluded that structured and consistent photoprotection significantly improves recovery and patient satisfaction (Level Ia).

For procedures with higher risk of PIH, such as fractional CO_2_ or picosecond laser in patients with skin of color, sunscreens containing additional anti-inflammatory agents (e.g., niacinamide, panthenol) may further mitigate pigmentary complications [31,32]. Tinted sunscreens with iron oxide provide additional protection against visible light, particularly high-energy blue light, which is implicated in persistent pigmentation via opsin-3-mediated pathways [51,52]. These formulations also improve cosmetic acceptability by camouflaging erythema and dyschromia, thereby encouraging adherence. For this reason, tinted or iron oxide-containing sunscreens are increasingly considered best practice in patients with darker phototypes.

Selection of sunscreen formulations should therefore prioritize broad-spectrum physical products containing zinc oxide and/or titanium dioxide with SPF ≥ 30, while reserving chemical sunscreens with caution in the early post-procedure phase to avoid sensitization [14,53]. Patients should be instructed on proper application techniques, including generous coverage of all treated areas and reapplication every 2–3 h when outdoors. However, data by Ruvolo et al. [44] suggest that very water-resistant sunscreens may provide sustained protection for up to six hours in low-exposure, indoor conditions. Thus, reapplication schedules may be tailored to individual lifestyle and risk, maintaining strict intervals in high-exposure settings but allowing extended duration for controlled indoor environments. Photoprotection should be continued daily for at least 3–6 months post-treatment, alongside protective clothing and behavioral sun avoidance during peak UV hours [32,45].

**Figure 1:**
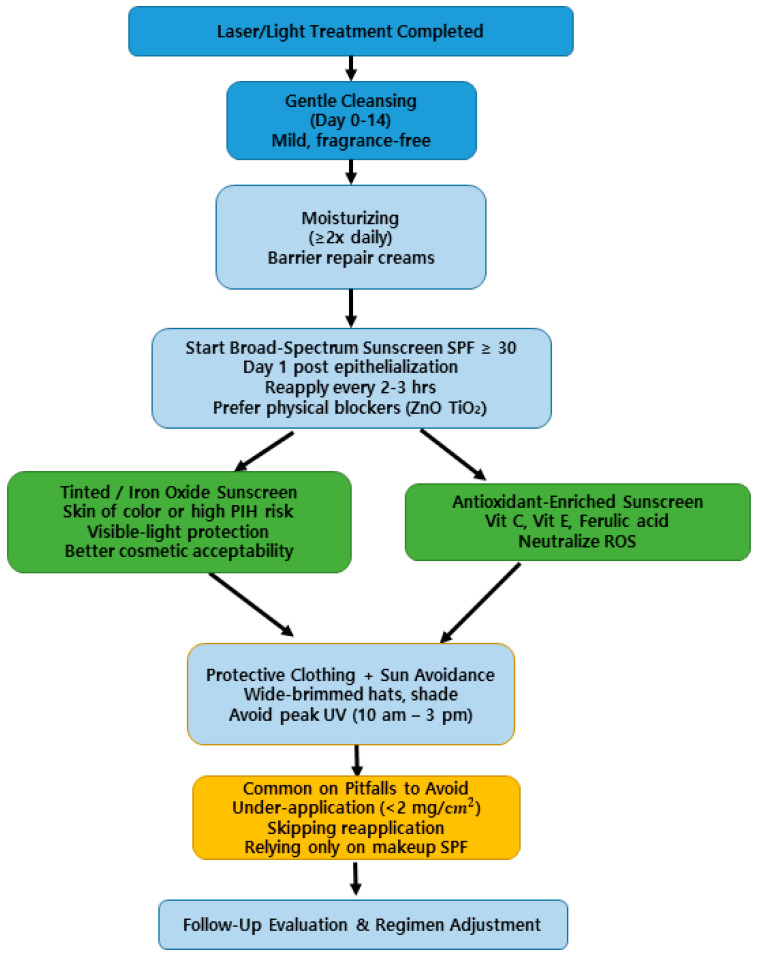
Flowchart of practical recommendations for post-laser photoprotection. Core steps (blue) include cleansing, moisturization, early initiation of broad-spectrum physical sunscreens, sun avoidance, and follow-up [24,30,53]. Optional measures (green) include tinted/iron oxide sunscreens for visible-light protection [51,52] and antioxidant-enriched formulations [30,44]. Common pitfalls (yellow) such as under-application or skipping reapplication are also highlighted [50].

Individualized post-procedure guidance that incorporates treatment type, patient phototype, and PIH risk profile will optimize outcomes. Clinicians should proactively discuss PIH risk, provide clear instructions on sunscreen use, and reinforce protocols at follow-up visits. Figure 1 presents a step-by-step flowchart summarizing the recommended regimen—including cleansing, moisturization, sunscreen initiation, optional use of tinted or antioxidant-enriched formulations, pitfalls to avoid, and follow-up—to improve patient comprehension and adherence.

To complement this, Table 2 summarizes key clinical studies stratified by laser type, patient phototype, sunscreen formulation, and outcomes, while Table 3 presents a concise “Do & Don’t” checklist for practitioners managing post-laser care. Together with Table 1 (overview of active sunscreen ingredients and Oxford Levels of Evidence), these visual and tabular resources provide clinicians with quick, evidence-based reference tools for daily practice.

### 4.5. Future Research Directions

Future research should prioritize well-designed, head-to-head clinical trials directly comparing physical and chemical sunscreen formulations in patients during the immediate post-laser recovery phase. Such studies should assess both objective clinical endpoints—such as erythema reduction, PIH prevention, and healing time—and patient-reported outcomes including cosmetic acceptability, texture, and ease of application [14,53]. In parallel, real-world studies are needed to better understand adherence patterns in diverse populations, as sunscreen use in post-procedural care remains suboptimal due to cosmetic dissatisfaction, perceived inconvenience, and regimen complexity [24,30]. Interventional trials should evaluate targeted strategies to improve compliance, including patient-centered education, simplified photoprotection regimens, and the development of cosmetically elegant, combination formulations [28,29]. Addressing these areas will not only strengthen the evidence base for sunscreen selection post-laser but also bridge the gap between controlled clinical settings and real-world patient behavior, ultimately enhancing clinical outcomes and patient satisfaction.

## 5. Conclusions

Integrating sunscreen use into post-laser facial treatment care is critical for optimizing patient outcomes and preserving the benefits of these procedures. Laser treatments, while effective in enhancing skin appearance and addressing various dermatological concerns, can leave the skin vulnerable to UV radiation, leading to a heightened risk of adverse effects such as hyperpigmentation and prolonged healing. This underscores the necessity for patients to understand the importance of sun protection in the aftermath of such treatments.

Evidence from numerous studies indicates that consistent application of broad-spectrum sunscreen significantly mitigates the risks associated with UV exposure, ensuring both the safety and effectiveness of laser therapies. Sunscreens with an SPF of 30 or higher are recommended, as they provide adequate protection and can help prevent the complications that diminish treatment results. Additionally, regular reapplication is essential, particularly in instances of prolonged sun exposure, to maintain this protective barrier.

Furthermore, patient education plays a vital role in promoting adherence to sunscreen protocols. Clinicians must prioritize discussing the importance of sun protection during consultation and follow-up visits, reinforcing the message that diligent sunscreen use is an integral part of the laser treatment journey. Empowering patients with knowledge and practical guidance helps foster a proactive approach to their skin care, enhancing their overall treatment experience.

Future research should continue to explore the long-term impacts of sunscreen application on the outcomes of various laser treatments, as well as any emerging technologies in sun protection. By emphasizing the critical role of sunscreen in post-laser care, healthcare professionals can improve patient satisfaction, prolong the effects of laser treatments, and ultimately enhance the standard of care within aesthetic dermatology. The adoption of comprehensive sun protection strategies is essential not only for patient safety but also for maximizing the aesthetic benefits derived from laser therapies.

Incorporating a thematic synthesis of findings across studies enhances the clarity and clinical utility of this review. Grouping evidence based on laser type, sunscreen formulation, and patient skin phototype allows for a more structured understanding of outcomes. For example, studies evaluating CO_2_ laser resurfacing consistently highlight the increased importance of photoprotection in minimizing post-inflammatory hyperpigmentation [12,19]. Likewise, the integration of sunscreens containing anti-inflammatory agents, such as niacinamide and panthenol, has been associated with reduced erythema and accelerated healing [24,26]. A concise summary table of active sunscreen ingredients, their mechanisms of action, and clinical benefits can assist practitioners in tailoring post-laser care recommendations to patient-specific needs. This approach not only synthesizes the available evidence but also provides actionable guidance for improving patient adherence and long-term treatment outcomes.

## Figures and Tables

**Table 1:** Active ingredients in sunscreens with anti-inflammatory properties and their mechanisms.

Active Ingredients	Mechanism of Action	Clinical Benefit	References
Niacinamide (Vitamin B3)	Inhibits inflammatory mediators, reduces transepidermal water loss, enhances skin barrier function	Reduces erythema, improves barrier recovery post-laser treatment	Marques et al., 2024 [39]; Boo et al., 2024 [40]
Panthenol (Provitamin B5)	Converted to pantothenic acid; promotes epithelialization, has humectant and anti-inflammatory effects	Accelerates wound healing, soothes irritation	Cho et al., 2022 [41]
Aloe vera extract	Contains polysaccharides and glycoproteins that modulate inflammatory pathways	Reduces erythema, promotes healing	Catalano et al., 2024 [42]
Glycyrrhetinic acid (from licorice root)	Inhibits cyclooxygenase activity and prostaglandin E2 formation	Decreases redness, calms sensitive skin	Kowalska et al., 2019 [43]

**Table 2:** Summary of key studies on sunscreen use after laser and light-based therapies with corresponding Oxford Levels of Evidence.

Study (Year)	Laser/Light Type	Patient Skin Phototype	Sunscreen Type/Composition	Key Outcomes	Level of Evidence
Fulton et al. (1998) [24]	CO_2_ Laser Resurfacing	II–IV	Broad-spectrum physical SPF ≥ 30	Reduced hyperpigmentation, erythema prevention	IV
Wanitphakdeedecha et al. (2014) [30]	Ablative Fractional Laser	III–V	Physical SPF 50 (zinc oxide + titanium dioxide)	Faster recovery, reduced inflammation	IIb
Puaratanaarunkon & Asawanonda (2022) [31]	Picosecond Laser	III–IV	Broad-spectrum + anti-inflammatory (niacinamide)	Lower PIH incidence	Ib
Passeron et al. (2019) [45]	Fractional/Q-switched Lasers	III–V	Tinted physical sunscreen + iron oxide	Reduced visible light-induced pigmentation	IIIa
Jones et al. (2018) [46]	IPL	II–III	Broad-spectrum SPF 50	Reduced erythema, improved skin hydration	IV
Tran et al. (2021) [49]	Ablative Resurfacing	II–IV	Broad-spectrum physical SPF ≥ 30	Immediate initiation reduced PIH	Ib
Xu et al. (2022) [50]	Multiple modalities (systematic review)	Mixed	Broad-spectrum photoprotection	Consistent sunscreen use improved recovery & patient satisfaction	Ia
López et al. (2020) [51]	Observational (Skin of color, multiple procedures)	IV–VI	Tinted sunscreen with iron oxides	Improved visible-light protection, better adherence in darker phototypes	IIIa
Tuchinda et al. (2021) [52]	Mechanistic & clinical studies	III–V	Iron oxide–based tinted formulations	Reduced blue light-induced pigmentation (opsin-3 pathway)	IIa

**Table 3:** “Do & Don’t” list for post-laser sunscreen guidance.

Do	Don’t
Use broad-spectrum SPF ≥ 30 from Day 1 [24,30,49]	Delay sunscreen application until redness subsides
Prefer physical blockers (zinc oxide, titanium dioxide) in early healing phase [14,53]	Use fragranced or alcohol-based sunscreens immediately post-procedure
Reapply every 2–3 h when outdoors [14,44]	Assume one morning application lasts all day
Consider tinted/iron oxide sunscreens for skin of color [45,51,52]	Ignore visible light as a pigmentation trigger
Combine with anti-inflammatory ingredients (niacinamide, panthenol) [31,32]	Overuse chemical filters that may cause irritation in compromised skin
Use antioxidant-enriched sunscreens (Vit C, E, Ferulic acid) [30,44,50]	Rely solely on sunscreen without other photoprotection measures (hats, shade)
Educate patients on PIH prevention [24,30,50]	Delay sunscreen application until redness subsides

## Data Availability

Not applicable.
